# Locked Nucleic Acid Oligonucleotides Facilitate RNA•LNA-RNA Triple-Helix Formation and Reduce *MALAT1* Levels

**DOI:** 10.3390/ijms25031630

**Published:** 2024-01-28

**Authors:** Krishna M. Shivakumar, Gowthami Mahendran, Jessica A. Brown

**Affiliations:** Department of Chemistry and Biochemistry, University of Notre Dame, Notre Dame, IN 46556, USA; kshivaku@nd.edu (K.M.S.); gmahendr@nd.edu (G.M.)

**Keywords:** LNA, *MALAT1*, *MENβ*, METTL16, RNA•LNA-RNA triple helix, triple helix

## Abstract

*Metastasis-associated lung adenocarcinoma transcript 1* (*MALAT1*) and *multiple endocrine neoplasia-β* (*MENβ*) are two long noncoding RNAs upregulated in multiple cancers, marking these RNAs as therapeutic targets. While traditional small-molecule and antisense-based approaches are effective, we report a locked nucleic acid (LNA)-based approach that targets the *MALAT1* and *MENβ* triple helices, structures comprised of a U-rich internal stem-loop and an A-rich tract. Two LNA oligonucleotides resembling the A-rich tract (i.e., A_9_GCA_4_) were examined: an LNA (L15) and a phosphorothioate LNA (PS-L15). L15 binds tighter than PS-L15 to the *MALAT1* and *MENβ* stem loops, although both L15 and PS-L15 enable RNA•LNA-RNA triple-helix formation. Based on UV thermal denaturation assays, both LNAs selectively stabilize the Hoogsteen interface by 5–13 °C more than the Watson–Crick interface. Furthermore, we show that L15 and PS-L15 displace the A-rich tract from the *MALAT1* and *MENβ* stem loop and methyltransferase-like protein 16 (METTL16) from the METTL16-*MALAT1* triple-helix complex. Human colorectal carcinoma (HCT116) cells transfected with LNAs have 2-fold less *MALAT1* and *MENβ*. This LNA-based approach represents a potential therapeutic strategy for the dual targeting of *MALAT1* and *MENβ*.

## 1. Introduction

**Figure 1:**
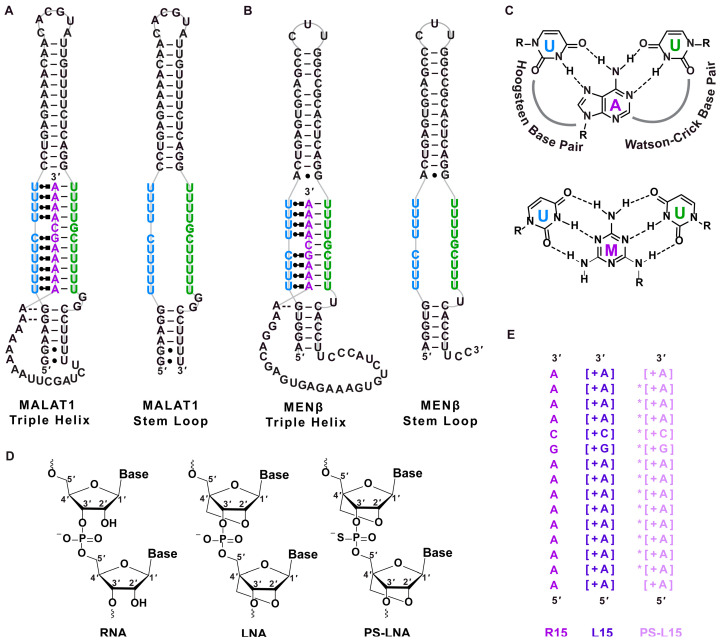
Structures of RNA, LNA and PS-LNA. Schematic diagrams of the (**A**) wild-type *MALAT1* triple helix and stem loop (SL), (**B**) wild-type *MENβ* triple helix and stem loop. The Watson–Crick and Hoogsteen interactions are represented by a solid line (|) and Leontis–Westhof notation (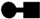) [48], respectively. (**C**) Chemical structures of U•A-U and U•M-U base triples with Hoogsteen and Watson–Crick base pairing denoted with dashed lines. The major-groove triple helix shows the Hoogsteen strand in blue and Watson–Crick strands in purple and green. (**D**) Chemical structure of ribose for RNA, LNA and phosphorothioate (PS)-LNA backbone. (**E**) Sequences of oligonucleotides R15, L15 and PS-L15. A + sign inside a square bracket [+] indicates locked nucleic acid sequence, whereas the asterisk (*) denotes phosphorothioate backbone. The colors purple, dark purple and light purple represent the R15, L15 and PS-L15 oligos, respectively.

Drugging RNA via small molecules and nucleic acids is an expanding field due to the vast size of the human transcriptome, particularly noncoding RNAs and RNAs directly linked to human health and disease [1,2,3,4]. Two such human long noncoding RNAs (lncRNAs), *metastasis-associated lung adenocarcinoma transcript 1* (*MALAT1*) and *multiple endocrine neoplasia-β* (*MENβ*)/*nuclear paraspeckle assembly transcript 1 (NEAT1)*, are highly conserved and non-essential RNAs whose abundance is correlated with many cancers, metastasis and poor patient outcomes [5,6,7,8,9,10]. Importantly, *MALAT1* is upregulated, most notably in leader cells, and the knockdown of *MALAT1* using antisense oligonucleotides (ASOs) reduces invasiveness in bladder cancer cells, as well as metastasis, organoid branching and the growth of the tumor in MMTV-PyMT (mouse mammary tumor virus–polyoma middle tumor antigen) mouse mammary carcinoma model [11,12]. A major reason for the high levels of *MALAT1* and *MENβ* is the unique 3′ ends of both lncRNAs, which feature a stem loop (SL) with a U-rich internal loop (Figure 1A,B), an A-rich tract and a tRNA-like structure, known as *MALAT1*-associated small cytoplasmic RNA (*mascRNA*) for *MALAT1* and *MENβ* tRNA-like small RNA (*menRNA*) for *MENβ* [13,14,15,16]. These tRNA-like structures are excised by ribonucleases (RNases) P and Z, leaving the mature *MALAT1* and *MENβ* RNAs with a 3′-triple-helical structure, in which the A-rich tract is sequestered by the U-rich internal loop and protects lncRNAs from degradation (Figure 1A–C) [13,14,15,16,17]. Inside the cell, *MALAT1* associates with methyltransferase-like protein 16 (METTL16), an *N*^6^-methyladenosine methyltransferase that requires the triple helix for binding but does not methylate it [18,19,20]. Because the triple helices of *MALAT1* and *MENβ* contribute to accumulation, these structures are potential drug targets [14,15,17]. 

Various approaches have been pursued to drug lncRNAs, including *MALAT1* and *MENβ*: small molecules, ASOs and bifacial peptide nucleic acids (bPNAs) mimicking the A-rich tract. The small molecules niclosamide and tyrphostin 9 reduce *MALAT1* levels via glycogen synthase kinase-3 beta (GSK3B) and heterogeneous nuclear ribonucleoproteins (hnRNPs) K and C [21]. However, most small molecules have targeted the *MALAT1* triple helix [22,23,24,25,26,27,28,29]. These small molecules belong to the classes of diphenylfuran derivatives [22,24], imidazole derivatives [23], diminazene derivatives [26], flavonoids [27] and aromatic heterocyclic compounds [25,29]. Although most small molecules have been shown to modulate the stability of the *MALAT1* triple helix in a test tube, the aromatic imidazole-derived compound **5** decreases endogenous *MALAT1* by 54% in MMTV-PyMT tumors and 38% in mammary tumor organoid branching, while *MENβ* levels do not change significantly [23]. Although the *MENβ* triple helix is less studied than its counterpart in *MALAT1*, compounds like aurintricarboxylic acid, emodin, GW5074, mitoxantrone and rottlerin bind to the *MENβ* triple helix near the micromolar range, and the kinase inhibitor PIK-75 abolishes paraspeckles in the neuroblastoma cell line SH-SY5Y [30]. ASO therapeutics have been used to target and regulate the *MALAT1* lncRNA in various cancer types [31,32,33,34,35,36,37,38,39,40,41,42,43,44,45]. A variety of ASOs, typically 16–20 nucleotides in length, have been designed, such as small interfering RNA (siRNA) [35,36,37]; gapmers with two to three locked nucleic acids (LNAs) [31,33,34,38,39,40]; 2′-O-methylethyl groups; 2′-O,4′-C-ethylene-bridged nucleic acid [41] or guanidine-bridged nucleic acid [42] at the end(s); PNA-DNA chimeras [43]; and the conjugation of ASO to single-wall carbon nanotubes [44], gold nanoparticles [32], TAT peptides [32], fatty acids [45] or membrane protein-binding aptamers [46] to improve delivery and biodistribution. Most ASO gapmers target unstructured regions of *MALAT1*, leading to RNase H-mediated knockdown from 2–50-fold [31,34,39,40]. For nucleic acid mimics or competitors, bPNAs have been synthesized with the nucleobase melamine, which can interact with the U-rich internal loop to form U•M-U base triples that are analogous to naturally occurring U•A-U base triples (Figure 1C). A bPNA targeting the SL region of the *MALAT1* triple helix showed an almost 50% reduction of *MALAT1* in pancreatic cancer cells [47]. One drawback is that the extremely short bPNA sequence of only four to six bases often experience off-target binding [47]. Overall, small molecules, ASOs and Xeno-nucleic acids (XNAs) have therapeutic value in decreasing *MALAT1* and *MENβ* levels in various cancer types.

Herein, we sought to target the SLs of *MALAT1* and *MENβ* RNAs using two LNA oligonucleotides, which we refer to as L15 and PS-L15. L15 has a ribose sugar, whereby a methylene bridge connects the 2′ oxygen and 4′ carbon to lock the sugar ring in a perfect C3′-*endo* conformation (Figure 1D) [49,50,51]. PS-L15 is an LNA with a phosphorothioate backbone, which can confer greater stability against enzymatic degradation than LNAs alone [52]. Additionally, LNAs can form tighter double- and triple-stranded structures than unmodified DNA and RNA [50,51,52,53,54,55,56,57]. We chose an all-LNA backbone for L15 and PS-L15 because triple helices prefer the C3′-*endo* conformation, theoretically creating more favorable binding conditions when an all-LNA oligonucleotide is the “middle” purine-rich strand of a triple helix [58]. L15 and PS-L15 are 15 nucleotides in length and have an A_9_GCA_4_ sequence (Figure 1E), an A-rich tract sequence that was previously shown to interact with the *MALAT1* and *MENβ* SLs [16]. Using L15 and PS-L15, our objectives were to determine if (i) a purine-rich all-LNA oligonucleotide could mediate both Hoogsteen and Watson–Crick interactions in the context of a pyrimidine-motif triple helix, (ii) the *cis*-acting A-rich tracts of *MALAT1* and *MENβ* could be displaced by LNAs, and (iii) LNAs could possibly function therapeutically by lowering levels of *MALAT1* and *MENβ* in cultured cells. Our experimental findings suggest that both L15 and PS-L15 form RNA•LNA-RNA triple helices upon binding to the *MALAT1* and *MENβ* SLs, preferentially stabilizing Hoogsteen base pairs more than Watson–Crick base pairs. L15 and PS-L15 can displace the A-rich tract and METTL16 from the *MALAT1* and *MENβ* triple helices, leading to a 2-fold reduction in *MALAT1* and *MENβ* levels in LNA-transfected human colorectal carcinoma (HCT116) cells. The 2-fold reduction in *MALAT1* levels via LNAs is comparable to compound **5**, a small molecule previously shown to target the *MALAT1* triple helix [23].

## 2. Results

### 2.1. LNA Oligonucleotides Bind to Both MALAT1 and MENβ SLs with Sub-Nanomolar to Nanomolar Dissociation Constants

**Figure 2:**
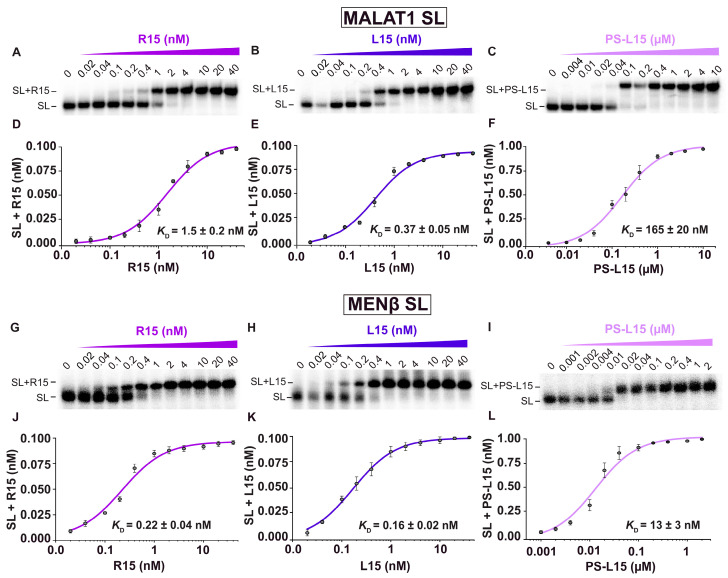
EMSA of SL and oligonucleotides. Representative gel images for the interaction between (**A**,**G**) *MALAT1*/*MENβ* SL and R15, (**B**,**H**) *MALAT1*/*MENβ* SL and L15 and (**C**,**I**) *MALAT1*/*MENβ* SL and PS-L15, showing a shift from stem loop to SL + oligonucleotide complex as increasing amounts of the respective oligonucleotides were added. Binding curves generated from the EMSA gel images for (**D**,**J**) *MALAT1*/*MENβ* SL and R15, (**E**,**K**) *MALAT1*/*MENβ* SL and L15 and (**F**,**L**) *MALAT1*/*MENβ* SL and PS-L15. The SL is visualized by 5’-[^32^P]-radiolabel. Each color denotes a specific oligonucleotide strand: purple for R15, dark purple for L15 and light purple for PS-L15. Reported *K*_D,app_ (apparent equilibrium dissociation constant) values are an average of at least three independent replicates and error bars represent standard deviation.

Previously, the R15 oligonucleotide, whose sequence is A_9_GCA_4_, was shown to interact with the *MALAT1* SL using a native electrophoretic mobility gel-shift assay (EMSA) [16]. To determine if the LNAs L15 and PS-L15 interact with the *MALAT1* SL, we employed a native EMSA to determine the apparent equilibrium dissociation constants (*K*_D,app_) (Figure 2, Table 1). The EMSA showed two distinct gel bands: one for the free SL and one for the oligonucleotide bound to the SL, presumably via the U-rich internal loop (Figure 2A–C). The *K*_D,app_ values obtained for the R15, L15 and PS-L15 binding to the *MALAT1* SL were 1.5 ± 0.2 nM, 0.37 ± 0.05 nM and 165 ± 20 nM, respectively (Figure 2D–F, Table 1). Despite the rigidity of an all-LNA backbone, L15 showed the tightest binding to the *MALAT1* SL. Because *MENβ* has a U-rich internal loop that can theoretically engage with L15 and PS-L15, we repeated the EMSA using the *MENβ* SL in the presence of R15, L15 and PS-L15 and determined *K*_D,app_ values of 0.22 ± 0.04 nM, 0.16 ± 0.02 nM and 13 ± 3 nM, respectively (Figure 2G–L, Table 1). The *MENβ* SL + oligonucleotide complexes are tighter than the *MALAT1* counterparts. We speculate that the shorter *MENβ* triple helix likely has greater flexibility to accommodate more optimal base triples (i.e., R•L Hoogsteen and L-R Watson–Crick base pairs) for the relatively inflexible all-LNA oligonucleotides [59,60,61].

Given the tight binding affinity, we next sought to determine if the LNAs could interact with any U-rich internal loop composed of nine or fewer Us; therefore, we used an EMSA to determine if the LNAs could bind to two other U-rich internal stem loops known to form triple helices: elements for nuclear expression (ENE) from Kaposi’s sarcoma-associated herpesvirus polyadenylated nuclear (KSHV *PAN*) lncRNA and double-domain ENE (dENE) from the *Oryza sativa* hAT-type DNA transposon *TWIFBIG1* (*TWIFB1*) (Figure S1A,B) [62,63]. No binding to the SLs was detected in the presence of up to 4 µM of R15, L15 and PS-L15 (Figure S1C,D). These results show that the LNAs L15 and PS-L15 can both bind to the *MALAT1* and *MENβ* SLs, showing selectivity for complementary Hoogsteen and Watson–Crick sequences.

### 2.2. L15 and PS-L15 Interact with MALAT1 and MENβ SLs via RNA•LNA-RNA Triple-Helix Formation

**Figure 3:**
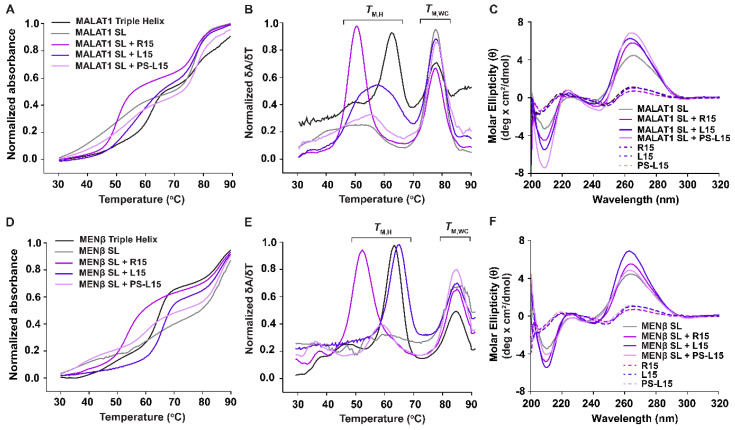
UV thermal melting and CD spectroscopy results showing profiles consistent with triple-helix formation. (**A**,**D**) Normalized absorbance versus temperature plot. (**B**,**E**) Normalized first derivative plot for the wild-type *MALAT1*/*MENβ* triple helix (black), *MALAT1*/*MENβ* SL (gray), *MALAT1*/*MENβ* SL + R15 complex (purple), MALAT/*MENβ* SL + L15 complex (dark purple) and MALAT/*MENβ* SL + PS-L15 (light purple). The brackets denote two distinct peaks: the Hoogsteen (T_M,H_) and the Watson–Crick (T_M,WC_) melting temperatures. (**C**,**F**) CD spectra are displayed as a plot of molar ellipticity (θ) versus wavelength. Single-stranded oligonucleotides R15 (purple), L15 (dark purple) and PS-L15 (light purple) are represented as dashed (--) lines. The *MALAT1*/*MENβ* SL (black) + oligonucleotide complexes for R15 (purple), L15 (dark purple) and PS-L15 (light purple) are represented as solid lines. The large differences in molar ellipticity at ~210 and ~270 nm indicate a change in structure when the single-stranded oligos (R15, L15 and PS-L15) are combined with *MALAT1*/*MENβ* SL, a characteristic of RNA triple-helical structures.

Based on our EMSA results, both L15 and PS-L15 interact with the *MALAT1* and *MENβ* SLs. Therefore, we next used UV thermal denaturation assays to probe if this interaction occurred via the formation of an RNA•LNA-RNA triple helix. First, triple-helix formation was tested for the *MALAT1* SL + R15 combination. As observed in previous studies [16,47,64], both the melting curves and first derivative plots show two distinct transitions/peaks, which correspond to the melting of Hoogsteen interactions (T_M,H_) at 49.8 ± 0.2 °C and Watson–Crick interactions (T_M,WC_) at 77.4 ± 0.1 °C (Figure 1C and Figure 3A,B, Table 2). For the *MALAT1* SL in the presence of oligonucleotides L15 and PS-L15, the T_M,H_ values were, respectively, 56.9 ± 0.2 °C and 54.6 ± 0.5 °C, whereas the T_M,WC_ values were 77.1 ± 0.1 °C and 77.3 ± 0.1 °C (Table 2). A similar biphasic melting profile was observed for the one-piece *MALAT1* triple helix, which is known to form an RNA triple helix [16,22,27,64] (Figure 1A and Figure 3A,B), whereas only one melting transition, which corresponds to T_M,WC_ at 77.5 ± 0.1 °C, was observed for the *MALAT1* SL, an RNA that cannot form a triple helix in the absence of an A-rich tract [16,27,47,64]. Thus, our UV melting curves suggest that the LNAs, despite their rigidity in a locked C3’-*endo* (north) conformation of the ribose sugar, are engaged with the SLs as a triple helix. Moreover, the Hoogsteen interface is more thermally stable for *MALAT1* SL + L15 and *MALAT1* SL + PS-L15 by 7.1 ± 0.2 °C and 4.8 ± 0.5 °C over *MALAT1* SL + R15 (Table 2). In contrast, the LNAs slightly destabilize the Watson–Crick interface (Table 2). However, the first derivative plots show noticeably different Hoogsteen peaks: tall and narrow for *MALAT1* SL + R15 but shorter and wider for *MALAT1* SL with L15 or PS-L15 (Figure 3B). This result suggests that there may be fewer fully formed base triples due to the structural rigidity associated with an all-LNA backbone in both L15 and PS-L15 [65,66,67].

The formation of a triple-helical structure was further probed using circular dichroism (CD) spectroscopy (Figure 3C). The CD spectrum of each oligonucleotide (i.e., R15, L15 and PS-L15) did not show any remarkable peaks, whereas the *MALAT1* SL displays a minimum at ~210 nm and maximum at ~265 nm, similar to the A-form duplex structure [27]. When the *MALAT1* SL is in the presence of the oligonucleotides R15, L15 and PS-L15, a larger difference in the molar ellipticity (θ), both at ~210 and ~265 nm, was observed in addition to weak maxima at ~220 nm and weak minima at ~244 nm. Strong peaks at 210 and 270 nm have been observed previously for RNA triple helices, including the *MALAT1* triple helix [27,57,68,69]. Finally, for both the UV and CD spectra, similar trends were observed when the *MENβ* SL bound to R15, L15 and PS-L15 (Figure 3D–F), except for two deviations: (i) the T_M,H_ and T_M,WC_ values are slightly greater than they are for the *MALAT1* counterparts, as observed previously [16], and (ii) the Hoogsteen peaks are consistently tall and narrow, suggesting a fully formed triple helix (Table 2). Altogether, our UV and CD spectroscopic results are consistent with the presence of a triple helix; therefore, we conclude that the LNA-based A-rich tracts can facilitate RNA•LNA-RNA triple-helix formation with the U-rich internal loops of both the *MALAT1* and *MENβ* SLs in a test tube.

### 2.3. LNAs Displace the A-Rich Tract and METTL16 from the MALAT1 Triple Helix

**Figure 4:**
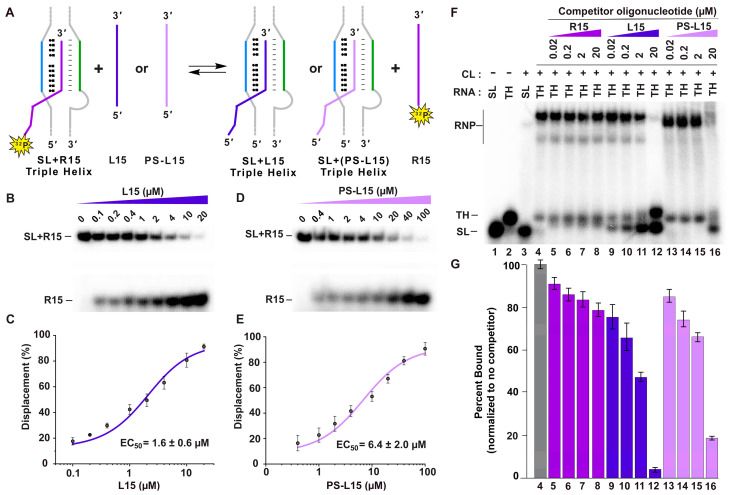
Competitive EMSA for the LNAs (L15 and PS-L15) displacing RNA (R15) from *MALAT1* SL + R15 complex and METTL16 (found in HCT116 cell lysate) from wild-type *MALAT1* triple helix. (**A**) Cartoon schematic showing the displacement of 5′-[^32^P]-radiolabeled R15 (purple) from SL + R15 complex by L15 (dark purple) or PS + L15 (light purple) to form SL + L15 or SL + (PS-L15) complexes. (**B**,**D**) Representative gel images and (**C**,**E**) binding curves for the displacement of R15 by L15 and PS-L15 from *MALAT1* SL + R15 complex. The gel images show dissociation of [^32^P]-radiolabeled R15 from SL + R15 complex as increasing amounts of L15 or PS + L15 are added. EC_50_ is the concentration corresponding to 50% displacement. (**F**) Representative gel image and (**G**) bar graph plot for the 5′-[^32^P]-radiolabeled *MALAT1* SL and *MALAT1* TH in the presence of HCT116 cell lysate (CL) and increasing amounts of competitor oligos R15 (purple), L15 (dark purple) and PS-L15 (light purple). RNP complex formed when METTL16 binds to *MALAT1* triple helix but not the *MALAT1* SL. The bar graph shows RNP complex formation normalized to no competitor, which was set to 100%. Error bars represent standard deviation from three independent runs.

Our next objective was to determine if the LNAs have any utility as a potential therapeutic, particularly for *MALAT1*. Inside cells, it is likely that the LNA will encounter a stable triple helix at the 3′ end of mature *MALAT1* or *MENβ*. Therefore, the LNA would have to be able to displace cis-acting A-rich tracts from the *MALAT1* and *MENβ* triple helices. To test if displacement is possible, we performed a competitive displacement assay (Figure 4A). Here, 5′-[^32^P]-radiolabeled R15 was folded with either the *MALAT1* or *MENβ* SL; then, increasing amounts of L15 or PS-L15 were added. For *MALAT1*, the experimentally determined EC_50_ values were 1.6 ± 0.6 μM and 6.4 ± 2.0 μM for L15 and PS-L15, respectively (Figure 4B–E, Table 1). Similarly, both LNA oligos displaced the 5′-[^32^P]-radiolabeled R15 from the *MENβ* SL, with EC_50_ values of 4.6 ± 1.0 μM and 11 ± 4 μM for L15 and PS-L15, respectively (Figure S2 and Table 1). These results demonstrate that the LNA oligos can potentially displace the A-rich tracts of *MALAT1* and *MENβ* inside cells. 

In a cellular environment, the *MALAT1* triple helix associates with METTL16 [18,19]. Therefore, the LNA oligos would have to be able to displace METTL16 in addition to the cis-acting A-rich tract of *MALAT1*. To determine if both displacements are possible, we performed a competitive EMSA using native HCT116 cell lysate. Whole-cell lysates were used because (i) the presence of the cellular milieu would be more physiologically relevant (i.e., more competitors), (ii) if there are other proteins binding besides METTL16, then those are accounted for, and (iii) as a stability element, the *MALAT1* triple helix is stable in cell lysates for the time period tested, so any RNase activity is negligible. Further, HCT116 is a cell line with a reasonable METTL16/*MALAT1* ratio, so a fully bound RNP is observable using a reasonable amount of whole-cell lysate. The competitive EMSA was set up using a 5′-[^32^P]-radiolabeled *MALAT1* triple helix in the presence of 25% cell lysate and with increasing concentrations of R15, L15 or PS-L15. As observed previously, no binding was detected for the *MALAT1* SL, but there was binding to the *MALAT1* triple helix (Figure 4F, lanes 3–4) [18]. The protein binding to the *MALAT1* triple helix is likely METTL16 and not non-specific binders (e.g., NPM, DHX9, ILF3, or ILF2) based on previous studies [18,19]. For the oligos, R15 showed minimal displacement at all concentrations (Figure 4F,G, lanes 5–8); however, 20 µM of L15 and PS-L15 showed a dramatic decrease, whereby less than 5% and 20% of the RNP complex remained, respectively (Figure 4F,G, lanes 12 and 16). Interestingly, LNA-induced displacement resulted in a band that migrated similarly to the SL control, suggesting that the displaced cis-acting A-rich tract was degraded up to the base of double-stranded stem (i.e., leaving the SL stably engaged with the LNA).

### 2.4. LNA Oligonucleotides Reduce MALAT1 and MENβ Levels in HCT116 Cells 

**Figure 5:**
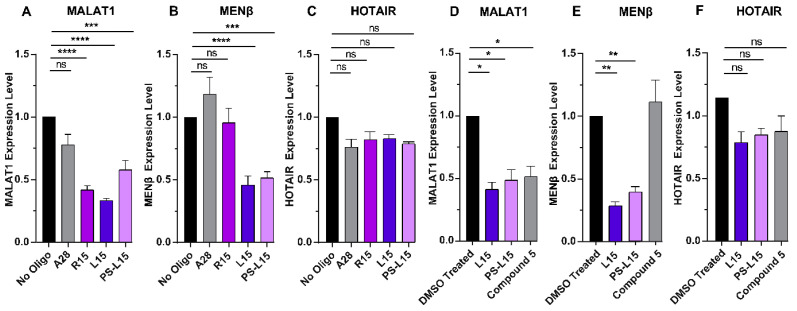
RT-qPCR results showing changes in expression for (**A**) *MALAT1*, (**B**) *MENβ* and (**C**) *HOTAIR* RNA when HCT116 cells were transfected with ~0.4 μM of A_28_, R15, L15 and PS-L15. RT-qPCR results showing changes in expression for (**D**) *MALAT1*, (**E**) *MENβ* and (**F**) *HOTAIR* RNA when HCT116 cells were transfected with 1 μM of L15 or PS-L15 or treated with 1 µM of compound **5**. The expression values were first normalized with respect to *U6 small nuclear* (*snRNA*) and these values were then normalized with respect to the no oligo/DMSO treated values set at 1. Results represent the mean ± SD of biological triplicates (n = 3). **** *p*-value < 0.0001, *** *p*-value < 0.0002, ** *p*-value < 0.0021 < * *p*-value < 0.033, ns = *p*-value < 0.1234 using two-way ANOVA test. Software used for statistical analysis was GraphPad Prism 8.0.1 (RRID:SCR_002798).

We next sought to explore how L15 and PS-L15 would alter *MALAT1* and *MENβ* levels inside the cells. HCT116 cells were transfected with R15, L15, PS-L15 or A_28_ as a non-complementary control, and then quantitative reverse transcription polymerase chain reaction (RT-qPCR) was employed to quantify the various RNA levels. Cells transfected with L15 or PS-L15 reduced *MALAT1* and *MENβ* levels by approximately 50%, but not the nuclear-localized long noncoding RNA *HOTAIR*, when compared to the cells with the mock transfection (Figure 5A–C). Because *MALAT1* and *MENβ* RNA levels are reduced in L15- and PS-L15-transfected cells, these results are consistent with the LNA oligonucleotides displacing the 3′-A-rich tract from a dynamic triple helix, making *MALAT1* and *MENβ* susceptible to RNA degradation by exonucleases. Such displacement would not be expected for the unmodified R15 (Figure 5A), unless the *MALAT1* triple helix is highly dynamic with an A-rich tract that rapidly dissociates from the U-rich internal loop [64]. 

Compound **5** (Figure S3) is a small molecule that was previously shown to specifically target the *MALAT1* triple helix [23]; therefore, we wanted to determine how effective the LNAs are at lowering *MALAT1* levels compared to compound **5**. HCT116 cells were transfected with equimolar amounts of L15, PS-L15 or treated with an equimolar amount of compound **5**. Our RT-qPCR results revealed that L15, PS-L15 and compound **5** showed a 50% reduction in *MALAT1* expression (Figure 5D). As reported previously, compound **5** reduces *MALAT1* but not *MENβ* despite the structural similarity of their triple helices (Figure 1A,B) [23]. In contrast, both L15 and PS-L15 exhibit a greater than 2-fold decrease in *MENβ* RNA in HCT116 cells (Figure 5E). L15, PS-L15 and compound **5** only mildly decrease the levels of *HOX antisense intergenic* (*HOTAIR*) RNA, but it is not statistically significant (Figure 5F).

## 3. Discussion

**Figure 6:**
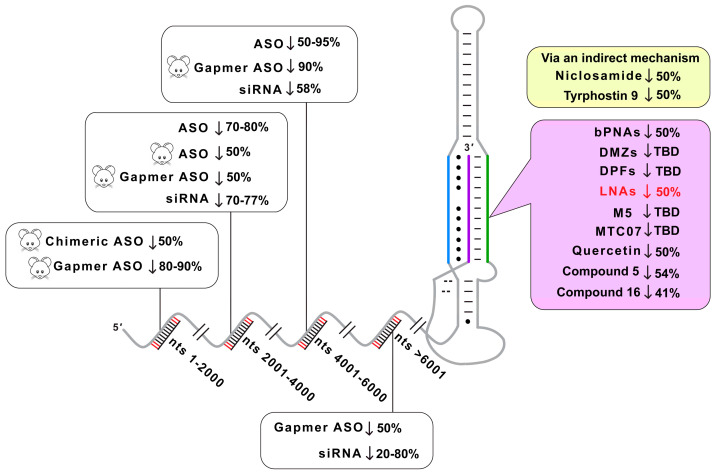
Summary of therapeutic strategies and effectiveness in lowering *MALAT1* levels. ASOs used to target human *MALAT1* include nts 2001–4000 [32,46], 4001–6000 [38,45] and nts 2001–4000 [38] on mouse *MALAT1*. Gapmer ASOs targeting human *MALAT1* nts 6001–8000 [40] and mouse *MALAT1* nts 1–2000 [34,42], 2001–4000 [39], 4001–6000 [38]. siRNAs used to target human *MALAT1* nts 2001–4000 [35,36], 4001–6000 [36], 6001–8000 [35,36]. Chimeric ASO used to target mouse *MALAT1* nts 1–2000 [43]. Niclosamide and tyrphostin 9 target *MALAT1* triple helix via an indirect mechanism [21]. Direct targeting of *MALAT1* triple helix by bPNAs [47], DMZs [26], DPFs [22], LNAs [this study], M5 [28], MTC07 [25], quercetin [27], compound **5** [23], and compound **16** [23]. To the best of our knowledge, the sequences of gapmer ASOs used to target *MALAT1* in Ref. [11] were not disclosed. A down arrow (↓) denotes the lowering of *MALAT1* levels, mouse cartoon denotes use of therapeutics in mice, TBD represents “to be determined” and red font denotes results from this study. Please note that the schematic extends beyond 8000 nts to account for ASOs targeting human *MALAT1*.

Ever since the overabundance of *MALAT1* and *MENβ* was correlated with various cancers, these lncRNAs have been targeted by small molecules as well as multiple nucleic acid-based agents (Figure 6) [1,2,3,4]. In this study, we examined an A-rich tract LNA to target the 3′-triple-helical structures critical to the stability of *MALAT1* and *MENβ*. Under the native gel-shift assay conditions tested, the overall binding trend was L15 > R15 >> PS-L15 for binding to the *MALAT1* and *MENβ* SLs (Figure 2). With *K*_D,app_ values in the sub-nanomolar range for the SL + L15 complexes, this binding outperformed small-molecule quercetin by 1000-fold and short oligos by 300-fold [22,23,26,27,47]. Oligonucleotides with at least one LNA base stabilizes the melting of DNA/RNA duplexes by 2–10 °C, which is one reason why LNAs are attractive therapeutic options [52,53,54]. For triple-helix structures, LNAs have been studied only in the Hoogsteen strand where they are known to greatly stabilize parallel Hoogsteen bonding, but not antiparallel reverse Hoogsteen bonding, with a complementary oligopurine target [55,56]. One study showed that LNA bases in the Hoogsteen strand of a pyrimidine-motif DNA triple helix (i.e., LNA•DNA-DNA) enhance the binding constant greater than 20-fold at neutral pH [55,56]. For RNA triple helices, LNA and 2-thiouridine modifications were incorporated into the Hoogsteen strand at various nucleotide positions. This LNA binding to a model RNA hairpin had a ΔT_M,H_ of 7–22 °C greater than when unmodified depending on the number and position of modified nucleotides, but the ΔT_M,WC_ was less than 4 °C [57]. 

To the best of our knowledge, our study is the first to investigate an RNA triple helix composed of an LNA-containing purine-rich strand, demonstrating that LNAs have a bifacial character by mediating both Hoogsteen and Watson–Crick interactions. The UV–thermal melting experiments of SLs + L15 or PS-L15 (Figure 3A,B,D,E) revealed that the Hoogsteen face is more thermally stable than the Watson–Crick face (Table 2), although the unfolding for the *MALAT1* SL + LNAs is generally less cooperative than it is for *MALAT1* SL + R15 (Figure 3B). Reduced cooperativity may correlate with the rigidity of LNA or geometric restrictions imposed by the chiral thiophosphate group [39,70]. Such rigidity may explain why *MENβ* forms a tighter RNA•LNA-RNA triple helix than *MALAT1* (Figure 2), although the persistence length of RNA triple helices is not yet known. Therefore, we do not know if the shorter *MENβ* triple helix is more flexible than the longer *MALAT1* triple helix (Figure 1A). However, both SLs can engage with an all-LNA oligo (Figure 2 and Figure 3), unlike an all-LNA triplex-forming oligonucleotide that failed to bind to complementary DNA and RNA duplexes [67]. For future investigations, it may be advantageous to design an LNA mixmer composed of natural and chemically modified nucleosides so that the increased flexibility can achieve greater stability of the *MALAT1* and *MENβ* RNA•LNA-RNA triple helices along the Hoogsteen and Watson–Crick interfaces.

Based on our competitive EMSA findings (Figure 4F,G), the displacement of the A-rich tract, METTL16 and any other protein binders from the *MALAT1* triple helix are important considerations, because 50% dissociation of L15 occurs around 2 µM [18,71]. bPNAs, on the other hand, show a slightly better displacement capability, with EC_50_ values ranging between 0.2 and 9 µM, albeit with a comparable reduction in the *MALAT1* expression levels of bPNA-treated pancreatic cancer cells [47]. Despite the tight complex that L15 and PS-L15 can form with the *MALAT1* SL (Figure 2B,C and Figure 3B,C), it is not strong enough to counteract the cellular degradation machinery, because our cell-based assays showed that the levels of *MALAT1* are reduced (Figure 5A). Additionally, compound **5** reduced *MALAT1* levels in HCT116 cells as much as L15 and PS-L15 (Figure 5D), even though the LNA oligos exhibit at least 100-fold tighter binding (Figure 2B,C,E,F) than compound **5** [23]. There are multiple factors that need to be considered when comparing results from cultured cells versus test tube. *MALAT1* is primarily nuclear, more specifically in nuclear speckles [5,6]. We know the efficacy of cellular delivery and local concentration of neither oligos nor compound **5** at the target site. Similarly, the half-life of the oligos versus compound **5** is also unknown. It is not clear to what extent the oligos or compound **5** would be sponged by non-specific binding partners. Deep-sequencing methodologies could be used to identify any non-specific targets of L15 and PS-L15. Another factor is the solubility and strength of binding in a liquid-phase condensate like nuclear speckles versus an aqueous environment in the test tube. The mechanistic details of A-rich tract displacement and LNA binding are unknown. Exonucleases may need only a small portion of the A-rich tract to be displaced, allowing degradation to occur before the LNA can fully engage with the *MALAT1* or *MENβ* SLs. Please note that an all-LNA oligo binding to *MALAT1* would be resistant to RNase H activity. Another consideration is the 3′-end processing of *MALAT1*, as the LNA could possibly bind to the U-rich internal loop before the triple helix forms at the end of mature *MALAT1* [13,72]. Such in vitro processing assays using reconstituted human RNase P and the RNA segment containing the stem loop, A-rich tract and tRNA-like structure have not been established. 

Both small molecules and nucleic acids reduce *MALAT1* and/or *MENβ* in cell-based assays, organoid systems and mouse models (Figure 6). Importantly, select ones show phenotypic effects, such as a smaller tumor size and slower cell proliferation. Small molecules like niclosamide, tyrphostin 9, imidazole-derived compound **5** and quercetin result in a 2-fold decrease in *MALAT1* levels upon treatment with various cancer cell types [21,23,27]. Both compound **5** and quercetin reportedly bind to the triple-helical region of the *MALAT1* triple helix, but not *MENβ*, despite their triple helices being structurally similar (Figure 1A,B) [23,27]. Furthermore, ASOs have been used to study and to target the *MALAT1* lncRNA in various cancer types. siRNAs against *MALAT1* display a 2-fold decrease in the *MALAT1* level, and its knockdown results in reduced cell migration in lung adenocarcinoma [35]. A nanocomplex carrying anti-*MALAT1* siRNA crosses the blood–brain barrier to target glioblastoma multiforme tumor cells, resulting in a 2–5-fold decrease in *MALAT1* levels, and induces increased sensitivity for temozolomide treatment [36]. In another report, an *siMALAT1* inhibits cell proliferation and promotes apoptosis by enhancing the expression of miR-145-5p in thymic cancer cells [37]. A short 16-mer ASO gapmer with phosphorothioate-modified S-2′-O-ethylene-2′,4′-bridged nucleic acid targeted *MALAT1* in an MMTV-PyMT carcinoma model, rendering slower tumor growth and reduced metastasis [11]. An LNA gapmer displays a greater than 50-fold decrease in *MALAT1* levels upon treatment in multiple myeloma cells, which is accompanied with reduced cell proliferation and increased apoptosis [31]. In a recent study, an LNA gapmer silenced 90% of *MALAT1,* in contrast to a 2′-O-methyl gapmer with 60% silencing when intratracheally administered to mice [34]. Most of the ASOs target the unstructured regions of *MALAT1*, leading to a 2–50-fold decrease in their level by recruiting RNase-H-mediated degradation and dysregulating its function in various cancer types [11,31,34,38,39,40]. In general, small molecules do not reduce *MALAT1* levels as much as some ASOs, although this deficiency could be overcome by ribonuclease targeting chimeras (RIBOTACS), a strategy that appends the small molecule to a heterocycle that recruits RNase L locally, thereby degrading *MALAT1* [73]. This RIBOTAC strategy could be applied to oligonucleotides like L15 and PS-L15 by adding aminothiophenone compounds [73]. Additionally, it may be possible to find small molecules that enhance the displacement of the A-rich tract, akin to what has been reported for small molecules displacing a strand of a DNA duplex [74]. A previous study examining bPNAs and this study examining LNAs demonstrate that the strand displacement of the A-rich tract leads to a 2-fold destabilization of *MALAT1* [47]. Because LNA mixmers typically bind tighter [52,53,54,65,66,67], it will be interesting to test L15 variants with an LNA backbone at only select locations. Unlike small molecules, modified oligonucleotides typically have poor cellular and tissue-specific delivery, limited endosomal escape and site-specific toxicity (e.g., kidney [75], neurons [76] and liver [77]) [78]. Our designed oligos would likely exhibit similar levels of toxicity as observed previously. However, one advantage of oligonucleotides over small-molecule therapeutics lies in the programmability of the nucleotide sequence, although the highly similar SLs of *MALAT1* and *MENβ* make it difficult to target only one of them. A therapeutic that simultaneously reduces the levels of both *MALAT1* and *MENβ* could be advantageous for cancers experiencing an upregulation of *MALAT1* and *MENβ*, such as lung, breast, colorectal and many other cancer types [9,10,79,80,81]. A previous study showed that gapmer and morpholino ASOs modulated the levels of paraspeckles, sub-nuclear bodies whose formation depends on *NEAT1* [82,83,84]. In theory, our oligos have the potential to disrupt and reduce paraspeckles because the levels of *NEAT1* decrease in HCT116 cells (Figure 5B,E). 

In summary, both L15 and PS-L15 show selective binding to the highly conserved *MALAT1* and *MENβ* SLs with sub-nanomolar to nanomolar dissociation constants [85]. This study demonstrates the ability of LNAs to recognize both the Watson–Crick and Hoogsteen faces of RNA SLs. Despite the greater stabilization seen with the Hoogsteen face over the Watson–Crick, it is unlikely that all the base triples adopt an optimal conformation due to structural rigidity of LNA, but this shortcoming might be overcome by using a mixmer. Keeping the advantages of LNAs over traditional DNA/RNAs, the current method establishes a simple, alternative approach to target the SL regions of the *MALAT1* and *MENβ* lncRNAs. 

## 4. Materials and Methods

### 4.1. RNA and Oligonucleotide Preparation

The oligonucleotides R15, L15, PS-L15 and A_28_ (Table S1) were purchased from Sigma-Aldrich (Woodlands, TX, USA) using the custom synthesis option, and stock solutions were prepared by dissolving the oligonucleotides in RNase-free deionized water. The *MALAT1* SL, *MALAT1* triple helix (TH), *MENβ* SL, *MENβ* triple helix, KHSV ENE and TWIFB1 dENE (Table S1) were prepared via in vitro transcription as previously described [16]. Homemade T7 RNA polymerase was used for the in vitro transcription and the resulting RNAs were gel-purified. Oligonucleotides or dephosphorylated in vitro-transcribed RNAs were 5′-end radiolabeled using γ-[^32^P]ATP (~7000 Ci/mmol, PerkinElmer, Boston, MA, USA) and T4 PNK (New England Biolabs, Ipswich, MA, USA) as per the manufacturer’s protocol. Excess γ-[^32^P]ATP was removed using G25 microspin columns (GE Healthcare, Buckinghamshire, UK).

### 4.2. Electrophoretic Mobility Shift Assays

Increasing amounts of oligonucleotides R15 or L15 (0–40 nM) were added to 0.1 nM of 5′-[^32^P]-radiolabeled *MALAT1*/*MENβ* SL, and increasing amounts of PS-L15 (0–400 nM) were added to 1 nM of 5′-[^32^P]-radiolabeled *MALAT1*/*MENβ* SL to maintain a good binding regime [86]. The 5′-[^32^P]-radiolabeled RNA and the oligo mixtures were folded in a previously reported binding buffer containing 25 mM of sodium cacodylate (pH 7.0), 50 mM of KCl, 1 mM of MgCl_2_ and 7.5% glycerol by heating at 95 °C for 5 min followed by slow cooling to room temperature for 1 h [16,87]. The reaction mixtures were further incubated for 30 min at 37 °C and then 48 h in a 4 °C cold room. A 48 h incubation period was used based on a previous study showing maximum binding of LNA after 24–48 h of incubation [88]. Unless specified, all samples were loaded onto a 12% native polyacrylamide gel (20 cm × 16 cm × 0.1 cm, 19:1 acrylamide/bisacrylamide, 40 mM of Tris-borate (pH 8.3), 1 mM of MgCl_2_) and electrophoresed with a running buffer (40 mM of Tris-borate (pH 8.3), 1 mM of MgCl_2_) at 220 V for ~6–8 h at 4 °C. For the *MENβ* SL with R15 and L15, 15% native polyacrylamide gels were prepared and electrophoresed at 220 V for 20–24 h at 4 °C. The gels were exposed to a Phosphorimager screen overnight after wrapping the gel in plastic wrap, scanned using an Amersham Typhoon IP Phosphorimager 1.0.0.7 (GE Healthcare, Tokyo, Japan) and analyzed using ImageQuant TL v8.1.0.0 software (GE Healthcare, Tokyo, Japan). A plot of the SL+oligonucleotide complex versus the concentration of oligonucleotides (R15, L15 and PS-L15) were fit to the quadratic equation (Equation (1)) using the OriginPro 2022 (64-bit) SR1 9.9.0.225 (Academic) graphing software (OriginLab Corporation, Northampton, MA, USA).

[complex] = 0.5(*K*_D,app_ + [SL] + [oligo]) − 0.5((*K*_D,app_ + [SL] + [oligo])^2^ − 4[SL][oligo])^0.5^ (1)

In Equation (1), [complex] refers to the concentration of the SL + oligonucleotide complex, [SL] is the initial *MALAT1*/*MENβ* SL concentration, [oligo] is the oligonucleotide (R15 or L15 or PS-L15) concentration and *K*_D,app_ is the apparent equilibrium dissociation constant. Here, the parameters [SL] and *K*_D,app_ were treated as variables. Please note that all binding curves were also fit to the Hill equation and the degree of cooperativity ranged from 1 to 1.5; therefore, we applied the quadratic equation as used previously for the *MALAT1* triple-helix two-piece RNA setup [16,87].

### 4.3. UV Thermal Denaturation Assay

UV thermal denaturation assays were conducted on a Cary 3500 Multicell UV-Vis Spectrophotometer (Agilent Technologies, Mulgrave, Australia) using quartz cuvettes (Starna Cells, Inc., Atascadero, CA, USA) with an optical path length of 1 cm. For each sample, the total RNA concentration (*MALAT1*/*MENβ* triple helix, *MALAT1*/*MENβ* SL with (1:1 stoichiometry) or without oligonucleotides) was maintained at 0.5 μM. Samples were prepared using a previously reported buffer containing 25 mM of sodium cacodylate (pH 7.0), 50 mM of KCl and 1 mM of MgCl_2_ [16]. RNA folding was completed by heating (25 °C to 95 °C) and cooling (95 °C to 25 °C) the samples at a ramp rate of 5 °C/min. After the folding step, the oligonucleotides were added and incubated for 30 additional minutes at 25 °C. For the melting curves, the absorbance at 260 nm was recorded at 0.3 °C intervals as the temperature increased from 25 °C to 95 °C at a ramp rate of 0.8 °C/min. The buffer was subtracted from all the melting curves of the RNA. The melting temperatures were extrapolated from the peak maxima of the first derivatives of the melting curves (δA/δT) and normalized between 0 and 1, followed by Savitzky–Golay smoothing across 25 points using the OriginPro 2022 (64-bit) SR1 9.9.0.225 (Academic) graphing software (OriginLab Corporation, Northampton, MA, USA).

### 4.4. Circular Dichroism Spectroscopy

CD spectra were obtained at 20 °C on a J-815 CD spectrometer (JASCO Corporation, Tokyo, Japan) using a 1 cm quartz spectrophotometer cell (Starna Cells, Inc., Atascadero, CA, USA). Individual solutions of oligonucleotides, *MALAT1*/*MENβ* SL and *MALAT1*/*MENβ* SL+oligonucleotides were prepared in a previously reported CD buffer composed of 25 mM of sodium cacodylate (pH 7.0), 125 mM of NaCl and 2 mM of MgCl_2_ [89]. The concentration of oligonucleotides and *MALAT1* SLs were fixed at 4 μM, whereby the SLs and oligonucleotides were mixed at 2 μM each (i.e., 1:1 stoichiometry). The samples were folded by heating at 95 °C for 5 min and slowly cooled to room temperature for 1 h, followed by incubation at 37 °C for 30 min. The samples were removed and placed inside a cold room at 4 °C for 48 h. For each spectral scan, the following parameters were used: 200–320 nm wavelength with continuous scanning mode, 4 s digital integration time, 2 nm bandwidth, 0.5 nm data pitch, standard sensitivity (±200 mdeg) and 100 nm/min scan speed. The data were an average of 5 scans, and molar ellipticity (θ) was calculated using Equation (2).

θ = mdeg × M/(10 × L × C) (2)

Parameters for Equation (2) are defined as follows: mdeg is the millidegree rotation measured by the CD spectrometer at a specific wavelength, M is the mean residual weight (i.e., the average molecular weight of nucleotide monophosphates in the oligonucleotides (324.53, 337.46, 352.46, 335.72, 333.67, 336.04, 338.78, 319.52 320.47, 322.93, 325.78 g/mol for R15, L15, PS-L15, *MALAT1* SL, *MALAT1* SL + R15, *MALAT1* SL + L15, *MALAT1* SL + PS-L15, *MENβ* SL, *MENβ* SL + R15, *MENβ* SL + L15, *MENβ* SL + PS-L15, respectively)), L is the path length (1 cm) and C is the molar concentration of nucleic acids in solution as individual nucleotide monophosphates (0.019, 0.020, 0.021, 0.089, 0.109, 0.110, 0.111, 0.081, 0.101, 0.102, 0.103, g/L) for R15, L15, PS-L15, *MALAT1* SL, *MALAT1* SL + R15, *MALAT1* SL + L15, *MALAT1* SL + PS-L15, *MENβ* SL, *MENβ* SL + R15, *MENβ* SL + L15, *MENβ* SL + PS-L15, respectively). The spectrum for buffer alone was subtracted from each sample spectrum. The data were an average of 5 scans and plotted as molar ellipticity versus wavelength.

### 4.5. A-Tract Displacement Assay

Two nanomolar of 5′-[^32^P]-radiolabeled R15 was mixed with 4 nM of the *MALAT1*/*MENβ* SL in a binding buffer containing 25 mM of sodium cacodylate (pH 7.0), 50 mM of KCl, 1 mM of MgCl_2_ and 7.5% glycerol. The RNA mixture was folded by heating to 95 °C for 5 min and snap cooling to 4 °C for 10 min, followed by incubation at room temperature for 1 h. Increasing concentrations of the oligonucleotides L15 (0–20 μM with *MALAT1* SL, 0–100 μM with *MENβ* SL) or PS-L15 (0–100 μM with each SL) were added and incubated at 37 °C for 30 min. Finally, the samples were removed and kept inside the 4 °C cold room for 48 h. The samples were loaded onto a 10% native polyacrylamide gel (19:1 acrylamide/bisacrylamide, 40 mM of Tris-borate (pH 8.3), 1 mM of MgCl_2_) and electrophoresed with the running buffer (40 mM of Tris-borate (pH 8.3), 1 mM of MgCl_2_) at 130 V for ~3 h at room temperature. The Saran-wrapped gels were exposed to a Phosphorimager screen overnight, scanned using an Amersham Typhoon IP Phosphorimager 1.0.0.7 (GE Healthcare) and analyzed using the ImageQuant TL v8.1.0.0 software (GE Healthcare, Tokyo, Japan). The % Displacement was calculated using Equation (3).

% Displacement = (I(SL + R15)/(I(SL + R15) + cI(R15))) × 100(3)

In Equation (3), I(SL + R15) is the band intensity for the SL + R15 complex, I(R15) is the band intensity for the radiolabeled R15 strand and ‘c’ is the constant obtained based on the ratio of the maximum band intensity for R15 over SL + R15. The EC_50_ values were determined by fitting the curve to the concentration of the agonist versus a response−variable slope model (Equation (4)) [90] using the OriginPro 2022 (64-bit) SR1 9.9.0.225 (Academic) graphing software (OriginLab Corporation, Northampton, MA, USA).

(4) $$\%\;\mathrm{Displacement}=a+[\mathrm{LNA}15{]}^{n}\;\times \;(b-a)/([\mathrm{LNA}15{]}^{n}+{EC}_{50}^{n}$$

In Equation (4), [LNA15] is the concentration of the LNA oligonucleotide (L15 or PS-L15) in micromoles and % Displacement is calculated using Equation (3). ‘a’ is the lower asymptote, the bottom of the curve or lower plateau (commonly referred to as the min), and ‘b’ is the upper asymptote, the top of the curve or upper plateau (commonly referred to as the max). The steepness of the linear portion of the curve is described by the slope factor, n. The parameter EC_50_ is the concentration corresponding to the response midway between ‘a’ and ‘b’.

### 4.6. Preparation of Native HCT116 Protein Lysate

HCT116 cells (RRID:CVCL_0291) were cultured at 37 °C with 5% CO_2_ in complete McCoy’s 5A (modified) media supplemented with 10% fetal bovine serum, 2 mM of glutamate and 1× penicillin–streptomycin. Cells (3 × 10 cm plates) were harvested when the confluency reached 80–90%. The pelleted cells were resuspended in 500 μL of the native lysate buffer (50 mM of Tris (pH 7) at room temperature, 100 mM of KCl, 0.2 mM of EDTA, 1 mM of MgCl_2_, 10% glycerol, 1 mM of DTT, 2% protease inhibitor cocktail (Sigma-Aldrich) and 1 mM of PMSF). The cells were sonicated (7 s, 3 times, with 30 s intervals) on ice and then centrifuged at maximum speed at 4 °C for 10 min. A BCA assay kit 23225 (Thermo Fisher Scientific, Waltham, MA, USA) was used for protein quantification and BSA as a standard. 

### 4.7. Competitive EMSA

Samples were prepared using 2 nM of 5′-[^32^P]-radiolabeled RNAs (*MALAT1* triple helix and *MALAT1* SL) in 1× EMSA buffer (25 mM of HEPES (pH 7.5) at room temperature, 50 mM of NaCl, 100 mM of KCl, 1 mM of TCEP, 1 mM of MgCl_2_, 7% glycerol and 1 mg/mL of yeast tRNA). The RNAs were folded by heating to 95 °C for 5 min and snap cooling on ice for 10 min, followed by equilibration at room temperature for 1 h. To the reaction mixture, increasing amounts of oligonucleotides (R15, L15, PS-L15) from 0 to 20 μM were added and incubated at 37 °C for 30 min. The samples were removed and kept inside a cold room at 4 °C for 48 h. An HCT116 native whole-cell lysate (~1 μg/μL unless indicated otherwise) was added to a binding mixture and incubated at room temperature for 30 min. The samples were loaded onto a 5% native polyacrylamide gel (19:1 acrylamide/bisacrylamide, 40 mM of Tris-borate (pH 8.3), 1 mM of MgCl_2_) and electrophoresed with the running buffer (40 mM of Tris-borate (pH 8.30, 1 mM of MgCl_2_) at 130 V for ~3 h at room temperature. The gel was exposed to a Phosphorimager screen overnight after wrapping the gel in plastic wrap, scanned using an Amersham Typhoon IP Phosphorimager 1.0.0.7 (GE Healthcare) and quantified with the ImageQuant TL v8.1.0.0 software (GE Healthcare, Tokyo, Japan).

### 4.8. Transfection of HCT116 Cells and RT-qPCR

HCT116 cells (RRID:CVCL_0291) were cultured at 37 °C with 5% CO_2_ in complete McCoy’s 5A (modified) media supplemented with 10% fetal bovine serum, 2 mM of glutamate and 1× penicillin–streptomycin. The cells were plated in a 24-well plate at a seeding density of 0.5 × 10^5^ cells/well and were allowed to reach ~70% confluency at the time of transfection. The Lipofectamine 3000 transfection reagent was diluted in an Opti-MEM reduced serum medium and 1 µg (or ~0.4 µM), which is the manufacturer’s recommended amount for the transfection of short oligonucleotides; R15, L15, PS-L15 or A_28_ were added to the cells. Mock transfected cells were treated with the same volume of McCoy’s 5A media. For cells treated with 1 µM of compound **5** (Sigma-Aldrich), compound **5** was dissolved in 100% DMSO (AmericanBio, Canton, MA, USA) and the final DMSO concentration was maintained at 0.1%. The cells were transfected with 1 µM of oligonucleotides. The cells did not show any morphological changes due to oligonucleotide transfection or treatment with compound **5**. Also, we did not observe any significant rate of cell death. Trizol (Life Technologies, Carlsbad, CA, USA) was used to extract RNA from the transfected and compound **5**-treated cells after 48 h. RNA pellets were resuspended in RNase-free water and then subjected to DNase treatment using RQ1 DNase (Promega, Madison, WI, USA, 1 U/μg of RNA), RQ1 buffer (1×) and an RNase inhibitor (Promega, 0.5 U/μL). The RNA was cleaned up using the Trizol method. First-strand cDNA synthesis was performed using random hexamer primers (50 pmol), a dNTP mix (0.5 mM), total RNA (5 μg) and the Superscript III reverse transcriptase (Invitrogen, Waltham, MA, USA). Previously published primers were used to quantify *MALAT1*, *MENβ*, *HOTAIR*, and *U6 snRNA* (Table S2) [18]. No template control and no reverse transcriptase control were performed. cDNA titration for *U6 snRNA* determined the amount of cDNA (1:10 dilution) to be used in the qPCR reaction. qPCR using the FastStart SYBR Green Master Mix (Sigma-Aldrich) was used with optimal cycling conditions (denaturation: 95 °C for 10 min, annealing: 60 °C for 1 min, extension: 72 °C for 1 min) using the QuantStudio 3 real-time qPCR instrument (Thermo Fisher Scientific, Marsiling, Singapore). 2^–ΔΔCt^ values [91] were calculated for the *MALAT1*, *MENβ* and *HOTAIR* RNAs from individual ΔCt values with normalization to *U6 snRNA*. The software used was GraphPad Prism 8.0.1. (RRID:SCR_002798).

## Figures and Tables

**Table 1:** Biophysical values for oligos R15, L15 and PS-L15 binding to *MALAT1* and *MENβ* SLs.

RNA/LNAs	*MALAT1* SL		*MENβ* SL	
	*K*_D,app_ (nM)	EC_50_ (μM)	*K*_D,app_ (nM)	EC_50_ (μM)
**R15**	1.5 ± 0.2	-	0.22 ± 0.04	-
**L15**	0.37 ± 0.05	1.6 ± 0.6	0.16 ± 0.02	4.6 ± 1.0
**PS-L15**	165 ± 20	6.4 ± 2.0	13 ± 3	11 ± 4

The values were obtained from three independent runs and represent average ± standard deviation. *K*_D,app_ is the apparent equilibrium dissociation constant and EC_50_ is the concentration corresponding to 50% displacement.

**Table 2:** Melting temperatures for the *MALAT1* and *MENβ* RNAs in the presence or absence of oligonucleotides.

RNA/LNAs	*MALAT1* RNA				*MENβ* RNA			
	*T*_M,H_	Δ*T*_M,H_	*T*_M,WC_	Δ*T*_M,WC_	*T*_M,H_	Δ*T*_M,H_	*T*_M,WC_	Δ*T*_M,WC_
**Triple Helix**	62.5 ± 0.3	-	77.2 ± 0.2	-	64 ± 0.1	-	85.5 ± 0.2	-
**SL**	-	-	77.5 ± 0.1	-	-	-	85.7 ± 0.2	-
**SL + R15**	49.8 ± 0.2	-	77.4 ± 0.1	-	52.6 ± 0.1	-	85.9 ± 0.3	-
**SL + L15**	56.9 ± 0.2	7.1 ± 0.2	77.1 ± 0.1	−0.3 ± 0.1	65.5 ± 0.5	12.9 ± 0.5	85.7 ± 0.2	−0.2 ± 0.2
**SL + PS-L15**	54.6 ± 0.5	4.8 ± 0.5	77.3 ± 0.1	−0.1 ± 0.1	59.2 ± 0.5	6.6 ± 0.5	86.0 ± 0.3	−0.1 ± 0.3

Melting temperatures for Hoogsteen and Watson and Crick transitions are, respectively, represented as *T*_M,H_ and *T*_M,WC_ in °C. Δ represents the change in melting temperature for SL + L15/PS-L15 – SL + R15 in °C. The values were obtained from triplicate runs and represent average ± standard deviation.

## Data Availability

Data are available upon request.

## Supplementary Materials

The following supporting information can be downloaded at https://www.mdpi.com/article/10.3390/ijms25031630/s1. References [23,48,62,63] are cited in the supplementary materials.
