# Supplementary material for: Locked Nucleic Acid Oligonucleotides Facilitate RNA•LNA-RNA Triple-Helix Formation and Reduce MALAT1 Levels
Source: Int J Mol Sci. 2024 Jan 28;25(3):1630. doi: 10.3390/ijms25031630 (PMC10855403; doi:10.3390/ijms25031630)
Supplement: Supplementary file 1 [file ijms-25-01630-s001.zip › ijms-2778484-supplementary.pdf]

SUPPLEMENTARY MATERIAL

Locked Nucleic Acid Oligonucleotides Facilitate RNA•LNA-RNA Triple-Helix Formation and Reduce *MALAT1* Levels

Krishna M. Shivakumar, Gowthami Mahendran, and Jessica A. Brown

**Table S1.** Oligonucleotides and RNAs used in this study.	
**Synthesized oligonucleotides**	
R15	5′-AAAAAAAAAGCAAAA-3′
L15	5′-[+A][+A][+A][+A][+A][+A][+A][+A][+A][+G][+C]<br>[+A][+A][+A][+A]-3′
PS-L15	5′-[+A]*[+A]*[+A]*[+A]*[+A]*[+A]*[+A]*[+A]*[+A]<br>*[+G]*[+C]*[+A]*[+A]*[+A]*[+A]-3′
A_{28}	5′-AAAAAAAAAAAAAAAAAAAAAAAAAAAA-3′
***In vitro* transcribed RNAs**	
MALAT1 SL	5′-GGAAGGUUUUUCUUUUCCUGAGAAAACAACACGUAUU<br>GUUUUCUCAGGUUUUGCUUUUUGGCCUUUU-3′
MALAT1 triple helix	5′-GGAAGGUUUUUCUUUUCCUGAGAAAACAACACGUAUU<br>GUUUUCUCAGGUUUUGCUUUUUGGCCUUUUUCUAGCUUAA<br>AAAAAAAAAAAGCAAAA-3′
MENβ SL	5′-AGGUGUUUCUUUUACUGAGUGCAGCCCAUGGCCGCACU<br>CAGGUUUUGCUUUUCACCUUCC-3′
MENβ triple helix	5′-AGGUGUUUCUUUUACUGAGUGCAGCCCAUGGCCGCACU<br>CAGGUUUUGCUUUUCACCUUCCCAUCUGUGAAAGAGUGAGCAGGAAA<br>AAGCAAAA-3′
KHSV *PAN* ENE	5′-GGCUGGGUUUUUCCUUGUUCGCACCGGACACCUCCAGU<br>GACCAGACGGCAAGGUUUUUAUCCCAGUUCGUAU-3′
TWIFB1 dENE	5′-GGCUGUACUCUUUUCUUUGUCAUGGUUUUCUCAAAUA<br>UGAGUUUUUACAUGACAAAGUUUUUAACGAGGCAGCA-3′
The "+" sign inside a square bracket [+] indicates locked nucleic acid modification, whereas the asterisk (*) denotes phosphorothioate backbone.	

**Table S2.** Primers used for RT-qPCR experiments.	
MALAT1	Forward: 5′-GATCTAGCACAGACCCTTCAC-3′<br>Reverse: 5′-CGACACCATCGTTACCTTGA-3′
MENβ	Forward: 5′-GTGTCCACAGGTCTTAGATTCC-3′<br>Reverse: 5′-TCTGTGTAGTAGGGTGGGATAG-3′
HOTAIR	Forward: 5′-GGTAGAAAAAGCAACCACGAAGC-3′<br>Reverse: 5′-ACATAAACCTCTGTCTGTGAGTGCC-3′
U6 snRNA	Forward: 5′-CTCGCTTCGGCAGCACA-3′<br>Reverse: 5′-AACGCTTCACGAATTTGCGT-3′

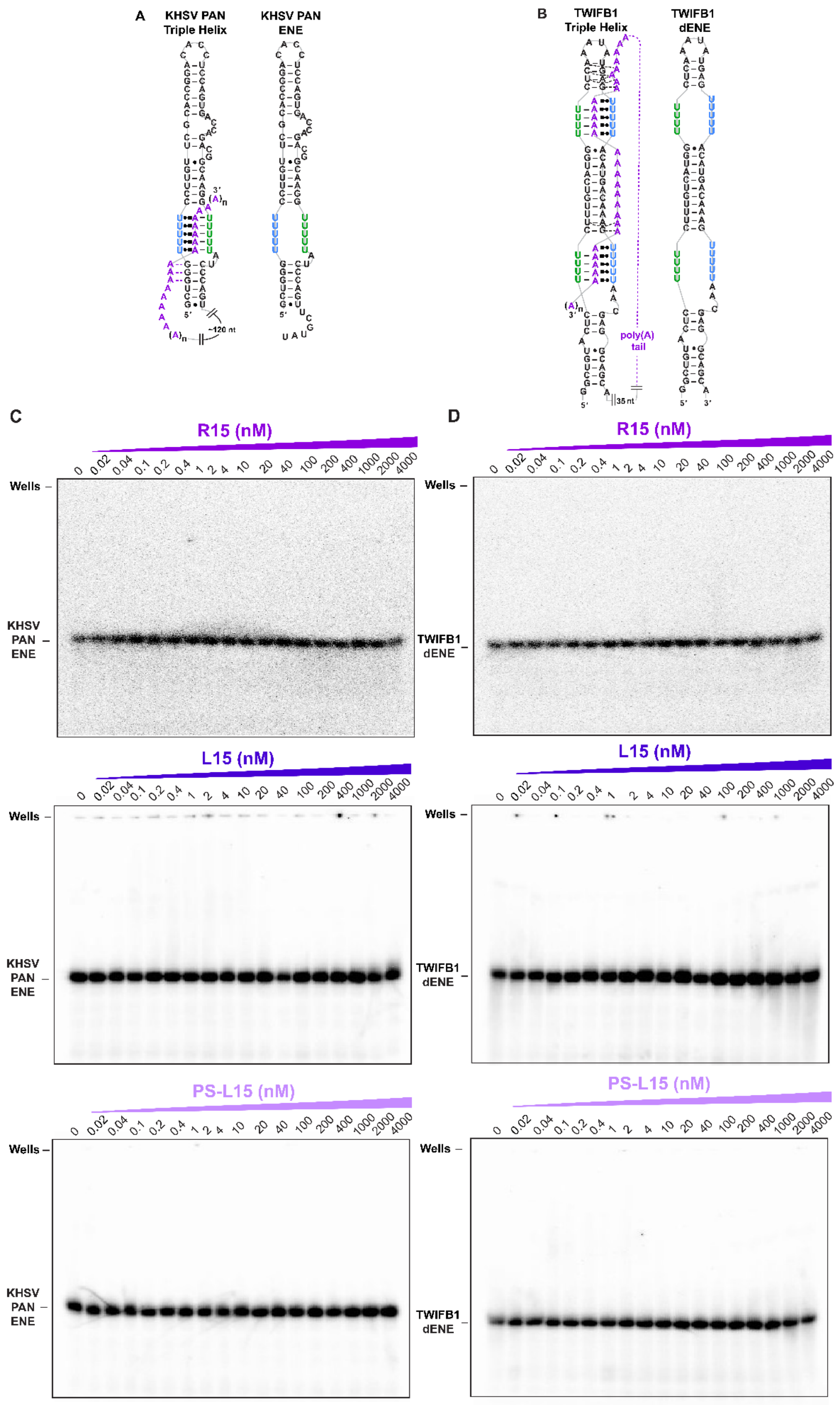
A
KHSV PAN Triple Helix
KHSV PAN ENE
~120 nt
B
TWIFB1 Triple Helix
TWIFB1 dENE
poly(A) tail
35 nt
C
D
R15 (nM)
0 0.02 0.04 0.1 0.2 0.4 1 2 4 10 20 40 100 200 400 1000 2000 4000
Wells
KHSV PAN ENE
TWIFB1 dENE
L15 (nM)
PS-L15 (nM)

Figure S1. EMSA for R15, L15 and PS-L15 not binding to the Kaposi's sarcoma–associated herpesvirus (KHSV) *PAN* ENE and mRNA element from *Oryza sativa* hAT-type DNA transposon *TWIFBIG1* dENE (*TWIFB1* dENE) RNAs [1,2]. Schematic diagrams of (**A**) KHSV *PAN* triple helix and its ENE, (**B**) rice transposase *TWIFB1* triple helix and its dENE. The Watson-Crick and Hoogsteen interactions are represented by a solid line (|) and Leontis-Westhof notation (●■) [3], respectively. Representative gel images for R15, L15 and PS-L15 titrated against (**C**) KHSV *PAN* ENE and (**D**) *TWIFB1* dENE.

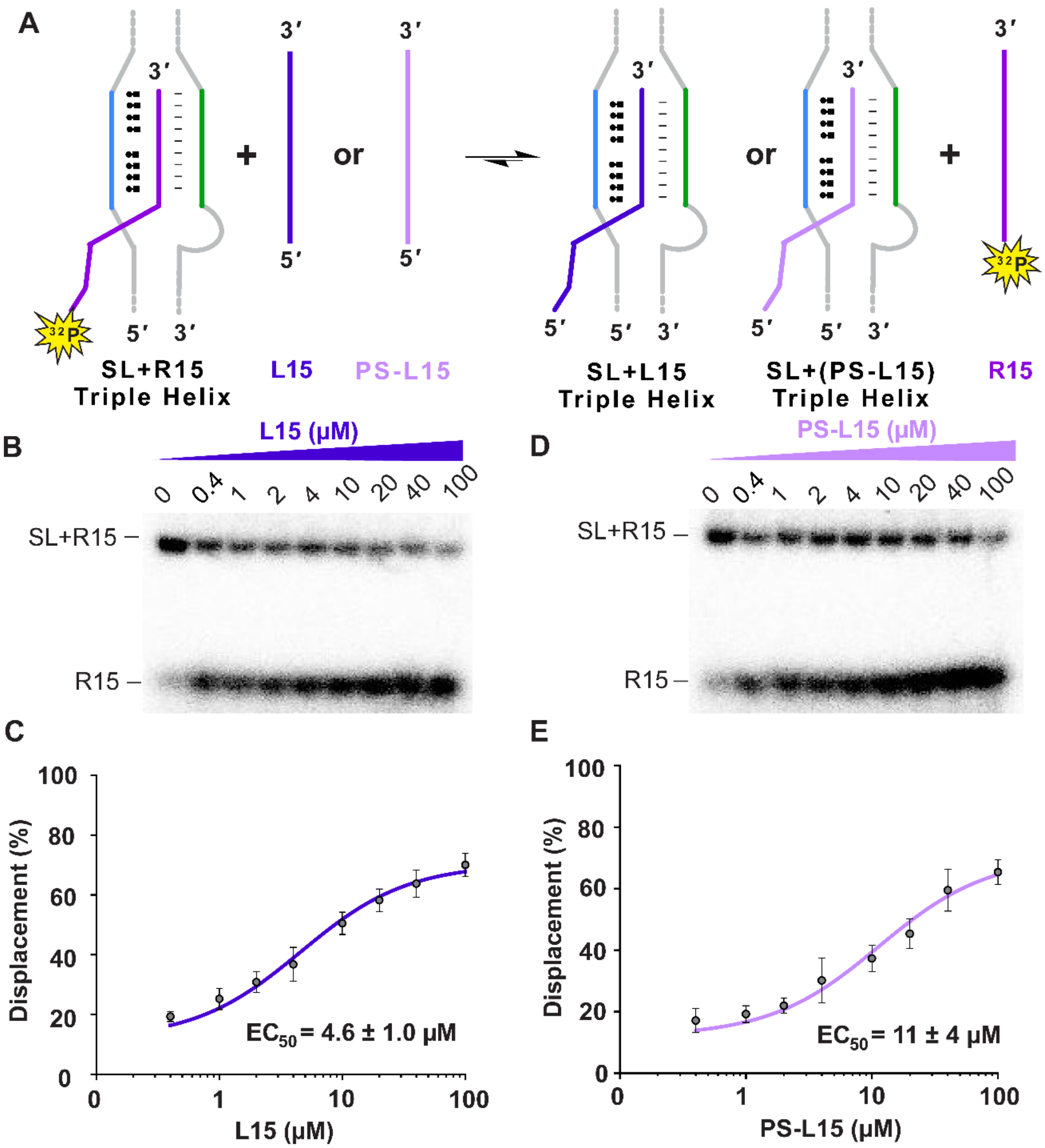

Figure S2. Competitive EMSA for the LNAs (L15 and PS-L15) displacing RNA (R15) from *MENβ* SL+R15 complex. (**A**) Cartoon schematic showing the displacement of 5'-[^{32}P]-radiolabeled R15 (purple) from SL+R15 triple helix by L15 (dark purple) or PS+L1 (light purple) to form SL+L15 or SL+(PS-L15) complexes. (**B, D**) Representative gel images and (**C, E**) binding curves for the displacement of R15 by L15 and PS-L15 from *MENβ* SL+R15 complex. The gel images show dissociation of 5′-[^{32}P]-radiolabeled R15 from SL+R15 complex as increasing amounts of L15 or PS+L15 are added.

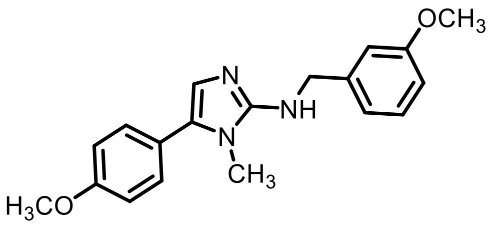

Figure S3. The chemical structure of compound **5**, a small molecule previously established to bind to the *MALAT1* triple helix [4].

REFERENCES

1. Mitton-Fry, R.M.; DeGregorio, S.J.; Wang, J.; Steitz, T.A.; Steitz, J.A. Poly(A) Tail Recognition by a Viral RNA Element Through Assembly of a Triple Helix. *Science* **2010**, *330*, 1244–1247, doi:10.1126/science.1195858.
2. Torabi, S.-F.; Vaidya, A.T.; Tycowski, K.T.; DeGregorio, S.J.; Wang, J.; Shu, M.-D.; Steitz, T.A.; Steitz, J.A. RNA Stabilization by a Poly(A) Tail 3′-End Binding Pocket and Other Modes of Poly(A)-RNA Interaction. *Science* **2021**, *371*, eabe6523, doi:10.1126/science.abe6523.
3. Leontis, N.B.; Westhof, E. Geometric Nomenclature and Classification of RNA Base Pairs. *RNA N. Y. N* **2001**, *7*, 499–512, doi:10.1017/s1355838201002515.
4. Abulwerdi, F.A.; Xu, W.; Ageeli, A.A.; Yonkunas, M.J.; Arun, G.; Nam, H.; Schneekloth, J.S.Jr.; Dayie, T.K.; Spector, D.; Baird, N.; et al. Selective Small-Molecule Targeting of a Triple Helix Encoded by the Long Noncoding RNA, MALAT1. *ACS Chem. Biol.* **2019**, *14*, 223–235, doi:10.1021/acschembio.8b00807.
